# Development of an anthropomorphic multimodality pelvic phantom for quantitative evaluation of a deep‐learning‐based synthetic computed tomography generation technique

**DOI:** 10.1002/acm2.13644

**Published:** 2022-05-17

**Authors:** Hyeongmin Jin, Sung Young Lee, Hyun Joon An, Chang Heon Choi, Eui Kyu Chie, Hong‐Gyun Wu, Jong Min Park, Sukwon Park, Jung‐in Kim

**Affiliations:** ^1^ Department of Radiation Oncology Seoul National University Hospital Seoul Republic of Korea; ^2^ Institute of Radiation Medicine Seoul National University Medical Research Center Seoul Republic of Korea; ^3^ Biomedical Research Institute Seoul National University Hospital Seoul Republic of Korea; ^4^ Department of Radiation Oncology Seoul National University College of Medicine Seoul Republic of Korea; ^5^ Robotics Research Laboratory for Extreme Environments Advanced Institute of Convergence Technology Suwon Republic of Korea; ^6^ Department of Radiation Oncology Myongji Hospital Goyang‐si Gyeonggi‐do Republic of Korea

**Keywords:** 3D printing, deep learning, magnetic resonance‐guided radiotherapy, multimodality phantom, synthetic CT

## Abstract

**Purpose:**

The objective of this study was to fabricate an anthropomorphic multimodality pelvic phantom to evaluate a deep‐learning‐based synthetic computed tomography (CT) algorithm for magnetic resonance (MR)‐only radiotherapy.

**Methods:**

Polyurethane‐based and silicone‐based materials with various silicone oil concentrations were scanned using 0.35 T MR and CT scanner to determine the tissue surrogate. Five tissue surrogates were determined by comparing the organ intensity with patient CT and MR images. Patient‐specific organ modeling for three‐dimensional printing was performed by manually delineating the structures of interest. The phantom was finally fabricated by casting materials for each structure. For the quantitative evaluation, the mean and standard deviations were measured within the regions of interest on the MR, simulation CT (CT_sim_), and synthetic CT (CT_syn_) images. Intensity‐modulated radiation therapy plans were generated to assess the impact of different electron density assignments on plan quality using CT_sim_ and CT_syn_. The dose calculation accuracy was investigated in terms of gamma analysis and dose‐volume histogram parameters.

**Results:**

For the prostate site, the mean MR intensities for the patient and phantom were 78.1 ± 13.8 and 86.5 ± 19.3, respectively. The mean intensity of the synthetic image was 30.9 Hounsfield unit (HU), which was comparable to that of the real CT phantom image. The original and synthetic CT intensities of the fat tissue in the phantom were −105.8 ± 4.9 HU and −107.8 ± 7.8 HU, respectively. For the target volume, the difference in *D*_95%_ was 0.32 Gy using CT_syn_ with respect to CT_sim_ values. The *V*_65Gy_ values for the bladder in the plans using CT_sim_ and CT_syn_ were 0.31% and 0.15%, respectively.

**Conclusion:**

This work demonstrated that the anthropomorphic phantom was physiologically and geometrically similar to the patient organs and was employed to quantitatively evaluate the deep‐learning‐based synthetic CT algorithm.

## 1 INTRODUCTION

The integration of on‐board magnetic resonance (MR) imaging with a radiotherapy treatment machine is an emerging technique for image‐guided radiotherapy.^1^, ^2^, ^3^, ^4^ Compared with computed tomography (CT),^2^ MR imaging affords superior soft tissue contrast. This can enhance the visibility of gross tumors and adjacent normal organs and improve the identification of the internal structure without exposing a patient to additional radiation. However, MR images do not provide the electron density information that is required for patient dose calculation.^3^

Several attempts have been made to utilize the electron density map in MR images for MR‐only radiotherapy.^5^, ^6^, ^7^, ^8^, ^9^, ^10^, ^11^ The MR images are fused with the CT image using the image registration algorithm for dose calculation. The use of this method is inevitable with the uncertainty of the image registration and the additional radiation exposure. Another approach is the conversion of MR intensity into a Hounsfield unit (HU), referred to as synthetic CT (sCT) or pseudo‐CT.^11^ Typically, an atlas‐based method is used for sCT generation.^12^ To match the atlas MR images, deformable registration is performed, followed by the conversion of the intensity to the HU information of the corresponding atlas CT images.

In learning‐based approaches, statistical learning or model fitting has been employed by training the relationship between CT and MR intensities.^11^, ^13^ Recently, a deep learning model has been developed for deriving highly accurate sCT estimation from MR images in near real time.^5^, ^14^, ^15^, ^16^, ^17^, ^18^, ^19^, ^20^ Han^5^ used a fully convolutional neural network to train the mapping from the MR images to corresponding CT images. To produce more realistic CT data, generative adversarial networks have been introduced with a discriminator distinguishing sCT images from real CT images.^15^ These techniques were evaluated by comparing sCT images with CT images that were deformed to MR images by the image registration algorithm. For accurate verification of deep‐learning‐based models, it would be ideal to collect data from real‐world clinical practice.^21^ However, the test dataset of the sCT model composed of deformed CT images inherently contained image registration uncertainty. Thus, it is necessary to quantitatively verify the sCT generation models with an independent dataset scanned from a standardized phantom that is free of deformation and applicable to both CT and MR scanners.

To date, only a few studies have fabricated multimodality phantoms applicable to CT and MR scanners.^22^, ^23^, ^24^, ^25^, ^26^, ^27^, ^28^ Sun et al.^28^ developed an MR‐compatible pelvic phantom for end‐to‐end testing procedures using the MR simulation process. They evaluated the geometric distortion using grid pattern parts and validated the simulation process with simplified models of human organs. Niebuhr et al.^25^, ^29^ investigated the tissue‐surrogate materials for MR and CT images and fabricated an anthropomorphic phantom for the MR‐based workflow. They used agarose gels with gadolinium‐based contrast agents and sodium fluoride (NaF) as the soft tissue surrogate. However, the NaF material caused imaging artifacts in the T2‐weighted MR images. Singhrao et al.^24^ developed an anthropomorphic pelvic phantom with carrageenan‐based materials to validate MR‐based workflows; this phantom allowed incorporation of film and ion chamber slots for radiation measurement. They quantified the MR and CT imaging characteristics of the phantom and evaluated the commercial sCT image generated by the scanned MR images. Although studies on the fabrication of multimodality phantoms have been previously performed, no investigation has ever evaluated a learning‐based sCT algorithm for MR‐only radiotherapy using a phantom. Deep‐learning‐based algorithms are trained using real patient image datasets; hence, it is necessary for the evaluation phantom to be similar to the patient in terms of geometry and imaging characteristics.

In this study, tissue‐surrogate materials combined with silicone oil were utilized as the MR signal modifier, and an anthropomorphic multimodality phantom was developed using a three‐dimensional (3D) printing technique. The sCT generation model was evaluated using the multimodality phantom by analyzing the intensity and dosimetric accuracy in an intensity‐modulated radiation therapy (IMRT) plan for prostate cancer.

## 2 MATERIALS AND METHODS

### 2.1 Material properties for phantom fabrication

To develop a multimodality phantom, the materials should be visible on both CT and MR images and should have similar image intensities to the patient's images.

The materials selected for the phantom are silicone‐based and urethane‐based materials, which are typically used for molding and coating in various applications. These materials are suitable for phantom fabrication because of their physical properties, that is, adequate hardness and durability. Four materials were tested for MR and CT compatibility: Dragon Skin 10 MEDIUM, VytaFlex 20, PMC‐780 DRY, and Smooth‐Cast 385 (Smooth‐on Inc., USA). Silicone oils of various viscosities (i.e., 100–3000 centistokes) at different concentrations were mixed to cure the phantom materials.

The materials were scanned using the MR‐guided radiotherapy system (ViewRay Inc., Cleveland, OH, USA) and Brilliance Big Bore CT simulator (Philips, Cleveland, OH, USA), which were employed for clinical radiation treatment planning. The materials were selected for phantom fabrication based on the evaluation of their MR and CT image characteristics.

### 2.2 Phantom design

A prostate cancer case was retrospectively selected for phantom construction. The CT scan for the phantom was conducted with a 120 kVp tube voltage and 1.5 mm slice thickness; it was reconstructed with a standard kernel. The same patient MR images were obtained using the 0.35 T ViewRay system. A true fast imaging with steady‐state precession (TrueFISP; TRUFI) sequence that yields a T_2_/T_1_‐weighted contrast was used for all MR scanning. A predefined field of view with an in‐plane resolution of 1.5 mm × 1.5 mm and a slice thickness of 1.5 mm was selected.

The images were anonymized and exported from the treatment planning system (TPS). The 3D‐Slicer, a free platform for biomedical research, was used to delineate the structures, that is, pelvic bone, prostate, bladder, rectum, soft tissue, and adipose tissue. The segmented structures were converted into the standard tessellation language (STL) format for 3D printing. The STL file was then modified using an open editing software program (Meshmixer, Autodesk); the model was hollowed out to form a 0.8‐mm‐thick structure wall. The phantom mold was manufactured from an amorphous thermoplastic material using a Zortrax M300 3D printer (Zortrax, Olsztyn, Poland). Following the 3D printing of the phantom mold, the support structures were removed, and selected materials were cast to each organ region. The phantom was placed in a pressure chamber to reduce bubble formation as the materials cured.

### 2.3 Generation of synthetic CT phantom images and validation of the image characteristics

The CT images (CT_sim_) of the phantom were acquired using the same CT simulator with the 120 kVp tube voltage, 200 mAs tube current–time product, 1.5 mm slice thickness, and standard kernel. The TRUFI MR images (MR_sim_) were acquired using the ViewRay scanner. The low‐frequency intensity and nonuniformity present in MR_sim_ were corrected using the N4 bias field correction algorithm.^30^ Synthetic CT phantom images (CT_syn_) were generated from a deep learning model using the MR_sim_ images. The sCT generation model trained in a previous study was used. The model consists of the 2D convolutional neural network, called U‐net by the shape of the learning structure. The details of the model hyperparameters and training datasets were described in the study.^14^ The CT_sim_, CT_syn_, and MR_sim_ imaging characteristics were quantified by measuring the CT HU and MR intensities of the prostate, bladder, soft tissue, adipose tissue, and cortical bone in the phantom. The region of interest (ROI) in the homogenous areas of target structures was defined to measure the mean and standard deviation values.

### 2.4 Validation of synthetic CT phantom images in treatment planning

The MR‐only simulation was performed using CT_syn_ to demonstrate the feasibility of the phantom in the quality assurance of treatment planning. The CT_sim_ and CT_syn_ were imported into the TPS of the ViewRay system, that is, the MRIdian system (ViewRay Inc.). We performed rigid registration from CT_sim_ to MR_sim_ to achieve electron density in the TPS. All contours in the MR_sim_ were delineated to define the normal organs and targets. To quantify the dosimetric analysis, the 3D dose distribution was calculated on the CT_sim_ images; then, the dose distribution of CT_syn_ images was calculated without changing the treatment plan parameters. The dose calculation was performed using the Monte Carlo dose algorithm of MRIdian TPS with magnetic field correction; the grid size was 3 mm, and the Monte Carlo uncertainty was 0.5%. Both CT_sim_ and CT_syn_ plans were normalized to include 95% of the target volume by at least 100% of the prescription dose.

For each of the plans, the dose‐volume histograms were extracted to derive the dose‐volume metrics. The mean, minimum, and maximum doses delivered to the target were calculated. The minimum doses (*D*_1%_, *D*_2%_, *D*_95%_, *D*_98%_, and *D*_99%_) that were delivered to at least 1%, 2%, 95%, 98%, and 99%, respectively, of each target volume between the CT_sim_ and CT_syn_ plans were calculated. For the bladder, the values of *D*_55%_, *D*_30%_, *D*_25%_, *V*_65Gy_, *V*_60Gy_, *V*_55Gy_, and the maximum dose were calculated. For the rectum, the values of *D*_50%_, *D*_20%_, *V*_65Gy_, *V*_60Gy_, *V*_55Gy_, and the maximum dose were calculated. The maximum dose for the femoral heads was also calculated.

## 3 RESULTS

### 3.1 Imaging properties of materials

Table 1 summarizes the intensities of the CT and MR images of the silicone‐based and urethane‐based materials mixed with various weight percentages of silicone oil. As the concentration of the silicone oil increased, the HU values approached that of the silicone. The viscosity of the silicone oil was not markedly affected by the CT intensities. In the case of the MR signal, the intensity tended to increase gradually as the weight percentage of the signal modifier for all materials increased. It was observed that the PMC‐780 material, that is, the polyurethane rubber compound, was not homogeneously cured with silicone oil because the viscosity of the latter was lower than that of the former.

**TABLE 1:** Testing materials of the multimodality phantom for magnetic resonance (MR) and computed tomography (CT) compatibility

CT number (HU)					
Material	Silicone oil viscosity	Weight percentage of signal modifier			
	(centistokes)	10%	20%	30%	40%
Dragon Skin 10 MEDIUM	3000	204.19	189.21	178.20	170.20
	1000	204.30	189.09	178.20	168.98
	500	204.00	188.36	177.00	167.75
	100	204.05	188.48	177.25	169.64
PMC‐780 DRY	3000	55.22	64.43	71.58	–
	1000	55.08	64.35	–	–
	500	55.08	63.87	–	–
	100	54.72	–	–	–
VytaFlex 20	3000	−0.11	14.74	19.86	27.25
	1000	−0.14	14.77	19.47	26.90
	500	0.08	14.99	20.47	26.94
	100	−0.48	14.28	17.88	25.14
MR intensity					
Dragon Skin 10 MEDIUM	3000	227.46	254.80	280.53	287.88
	1000	247.26	279.72	308.07	313.73
	500	238.22	265.64	301.92	315.73
	100	230.30	271.31	301.59	312.64
PMC‐780 DRY	3000	79.46	114.78	152.18	–
	1000	90.81	133.65	–	–
	500	89.18	130.48	–	–
	100	89.20	–	–	–
VytaFlex 20	3000	208.47	223.84	248.49	250.95
	1000	217.92	236.90	255.41	267.83
	500	197.60	221.43	244.85	249.31
	100	197.71	224.63	233.93	240.84

*Note*: The CT and MR intensities of the materials were measured using a CT simulator and 0.35 T MR scanner, respectively, because silicone oils of various viscosities were mixed with materials of different concentrations.

Abbreviation: HU, Hounsfield unit.

Based on the results, three materials were selected as tissue surrogates for the prostate, namely, soft tissue, urinary bladder, and spongy bone, by comparing the pixel intensities of the patient's image. In CT imaging, plastic‐based materials exhibit low signals; this is not suitable for parts with high attenuation, for example, cortical bone. The gypsum bandage, which can be an alternative for cortical bone, contains large amounts of calcium similar to human bone. For the adipose tissue simulation, olive oil with a hardening agent was selected as the tissue‐surrogate material. The selected materials for phantom construction are summarized in Table 2.

**TABLE 2:** Summary of materials for tissue surrogates

Tissue type	Tissue‐surrogate material	Silicone oil	
		Viscosity (centistokes)	Weight percentage (%)
Prostate	PMC‐780 DRY	1000	10
Soft tissue	PMC‐780 DRY	1000	10
Urinary bladder	VytaFlex 20	1000	40
Spongy bone	Dragon Skin 10 MEDIUM	1000	20
Cortical bone	Gypsum bandage	–	–
Adipose tissue	Olive oil, hardener	–	–

### 3.2 Validation of phantom imaging properties

**FIGURE 1:**
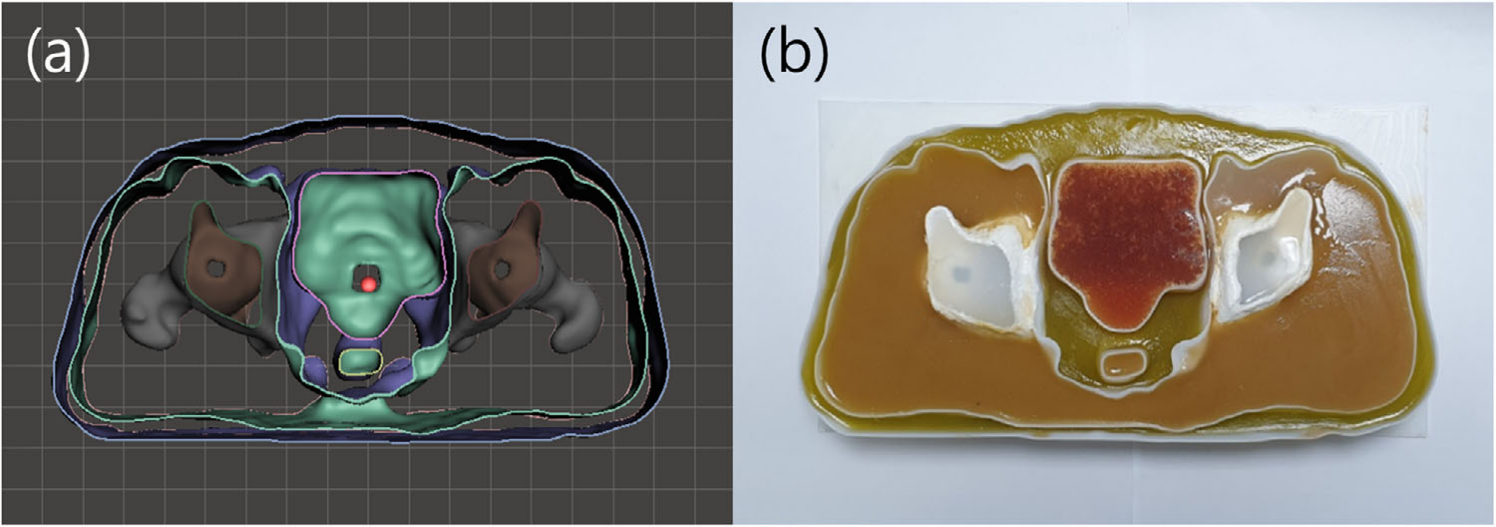
(a) The front of the phantom computer modeling for three‐dimensional printing and (b) the fabricated anthropomorphic pelvic phantom

**FIGURE 2:**
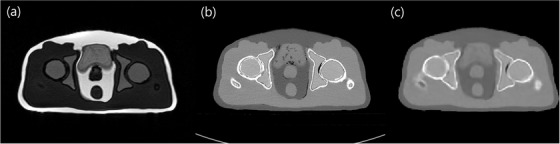
Phantom images scanned using (a) 0.35 T magnetic resonance (MR) with TRUFI sequence and (b) computed tomography (CT) simulator; (c) image derived from the image in (a) using synthetic CT generation model

The computer modeling for 3D printing and the fabricated pelvic phantom image is presented in Figure 1; the CT_sim_ and MR_sim_ images of the phantom are shown in Figure 2a,b, respectively. For comparison, the CT_syn_ image from the MR_sim_ is shown in Figure 2c.

The measured HUs of the phantom in both CT_sim_ and CT_syn_ and the corresponding MR_sim_ signal for several ROIs defined within the structures are summarized in Table 3; for comparison, the measured HUs of patient organs are also listed. The CT number for the prostate‐surrogate material was 28.5 ± 7.2 HU, whereas that for the patient was 38.9 ± 12.9 HU; the HU difference between CT_sim_ and CT_syn_ was 2.4. In the MR_sim_ images, the difference between the patient and phantom was 8.4. For the urinary bladder‐surrogate material, the measured CT numbers were 4.9 ± 30.6 HU and 6.8 ± 11.6 HU for the phantom and patient, respectively. The MR intensity of the bladder was 309.1 ± 17.8, comparable to that of the patient. In the case of spongy bone, the HU difference between the patient and phantom was relatively higher than that of other organ surrogates. However, a signal difference of only 4.4 was measured in the corresponding MR image. For the cortical bone, the CT numbers were 603.4 ± 186.4 HU and 626.4 ± 89.3 HU for the simulation CT and sCT, respectively. The MR signals measured in the region were 38.3 and 12.4, respectively. The measured CT number for the adipose tissue region of the phantom was −105.8 ± 4.9 HU. Only a 2.0 HU discrepancy was observed between the simulation CT and sCT images. The maximum absolute difference in the CT number between the simulation CT and sCT was 23.0 HU for the organ surrogates.

**TABLE 3:** Mean and standard deviations of computed tomography (CT) numbers and magnetic resonance (MR) intensities determined within defined regions of interest (ROIs) in patient, phantom, and synthetic CT phantom images of various anatomical regions

	CT number (HU)			MR intensity	
Organ	CT_patient_	CT_sim_	CT_syn_	MR_patient_	MR_sim_
Prostate	38.9 ± 12.9	28.5 ± 7.2	30.9 ± 10.1	78.1 ± 13.8	86.5 ± 19.3
Urinary bladder	6.8 ± 11.6	4.9 ± 30.6	17.5 ± 6.1	319.2 ± 17.7	309.1 ± 17.8
Spongy bone	379.8 ± 104.8	164.7 ± 9.0	145.8 ± 24.0	260.4 ± 49.1	256.0 ± 9.4
Cortical bone	740.4 ± 318.0	603.4 ± 186.4	626.4 ± 89.3	38.3 ± 9.1	12.4 ± 11.4
Soft tissue	48.9 ± 14.1	56.8 ± 7.3	51.0 ± 2.5	85.1 ± 39.8	94.3 ± 7.7
Adipose tissue	−93.2 ± 8.0	−105.8 ± 4.9	−107.8 ± 7.8	532.6 ± 25.1	607.4 ± 15.9

Abbreviation: HU, Hounsfield unit.

### 3.3 Dose–volume evaluation

**FIGURE 3:**
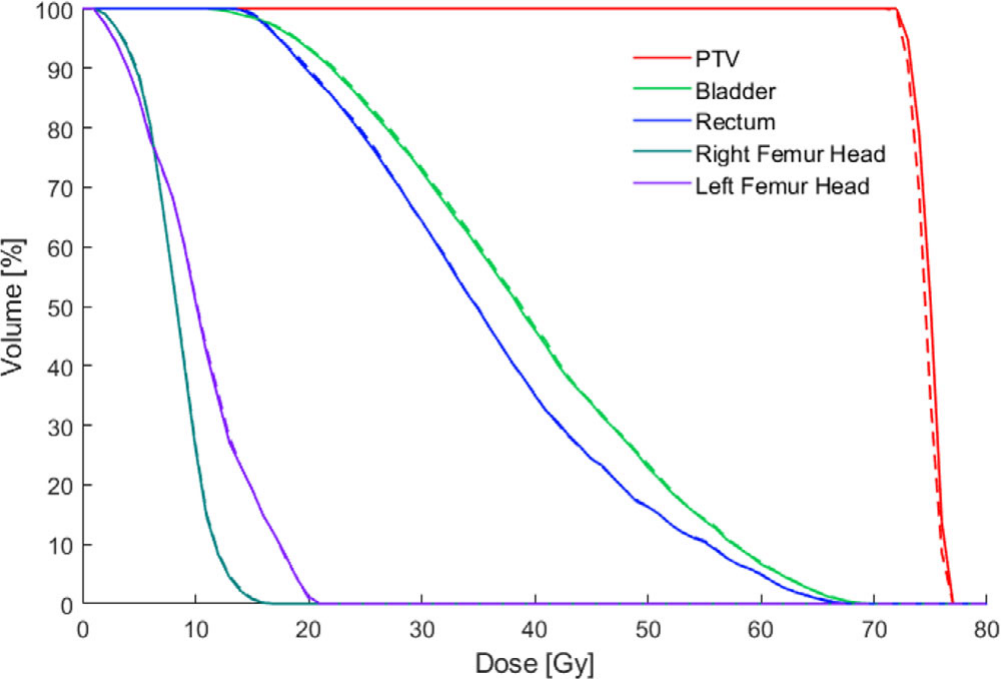
Comparison of dose–volume histograms between simulation computed tomography (CT_sim_) (solid line) and synthetic computed tomography (CT_syn_) (dashed line) plans

Dose–volume histogram curves for the planning target volume and organ at risk of the two IMRT plans are represented in Figure 3. Table 4 summarizes the dose‐volumetric parameters of the target and normal organs from the plans. For the target volume, the *D*_1%_, *D*_2%_, *D*_95%_, *D*_98%_, and *D*_99%_ of the simulation CT‐based plan were slightly higher than those of the sCT‐based plan. For the bladder, the *D*_55%_ and *D*_30%_ dose values and the percent volumes of the bladder receiving at least 65 Gy (*V*_65Gy_), 60 Gy (*V*_60Gy_), and 55 Gy (*V*_55Gy_) indicated that both the original IMRT plan and sCT‐based plan were acceptable. For the bladder, the maximum absolute difference among the dose‐volumetric parameters was less than 0.38 Gy. For the rectum, the *V*_65Gy_, *V*_60Gy_, and *V*_55Gy_ of the original plan were smaller than those of the sCT; however, the differences were less than 0.21%. The maximum doses for the bladder in CT‐based IMRT and MR‐based IMRT with sCT were 8.46 and 8.08 Gy, respectively. The gamma analysis of dose distribution in CT_sim_ and CT_syn_ within 2%/2 mm at a 10% dose threshold exhibited a 99.6% pass rate.

**TABLE 4:** Dose‐volumetric parameters of plans calculated based on simulation computed tomography (CT) and synthetic CT images of phantoms

	CT_sim_	CT_syn_	Difference
PTV			
*D*_1%_ (Gy)	76.83	76.74	0.09
*D*_2%_ (Gy)	76.36	76.23	0.13
*D*_95%_ (Gy)	69.68	69.36	0.32
*D*_98%_ (Gy)	68.88	68.60	0.28
*D*_99%_ (Gy)	68.48	68.15	0.33
Bladder			
Maximum dose (Gy)	8.46	8.08	0.38
*D*_55%_ (Gy)	29.96	30.00	−0.04
*D*_30%_ (Gy)	40.37	40.57	−0.20
*D*_25%_ (Gy)	43.26	43.22	0.04
*V*_65Gy_ (%)	0.31	0.15	0.16
*V*_60Gy_ (%)	3.04	2.90	0.14
*V*_55Gy_ (%)	8.00	7.85	0.15
Rectum			
Maximum dose (Gy)	10.96	11.32	−0.36
*D*_50%_ (Gy)	27.71	27.75	−0.04
*D*_20%_ (Gy)	36.93	36.95	−0.02
*V*_65Gy_ (%)	0.27	0.23	0.04
*V*_60Gy_ (%)	1.35	1.37	−0.02
*V*_55Gy_ (%)	4.39	4.18	0.21
Femoral heads			
Maximum dose (Gy)	7.86	8.10	−0.24

Abbreviations: *D_n_*_%_, highest dose received by at least *n*% volume of a structure; PTV, planning target volume; *V_n_*_Gy_, percent volume receiving *n* Gy.

## 4 DISCUSSION

In this study, a multimodal anthropomorphic phantom with a compatible tissue surrogate was designed and fabricated. The quantitative imaging characteristics of the phantom compared with those of the patient case were further analyzed. Finally, the application of the phantom for the evaluation of the deep‐learning‐based sCT image generation technique with the IMRT plan verification procedures was demonstrated.

In some studies related to tissue‐equivalent MR phantoms, a tissue‐equivalent material using agarose gel was produced.^24^, ^25^, ^31^, ^32^, ^33^ The relaxation time could be adjusted by changing the concentrations of agarose and paramagnetic ions. Agarose gel was also used for the ultrasound phantom because of its elastic and acoustic impedance characteristics.^34^ However, the typical problem encountered in the use of agarose gel materials was water loss, which led to changes in the physical properties.^34^ This problem could be solved using polymers made of siloxane or urethane; these materials could achieve long stability similar to that of a hard solid. Steinmann et al.^22^ tested the reliability of silicone‐based and urethane‐based materials by storing them at room temperature for 3 months; the materials exhibited no form of degradation. Hence, with the use of the foregoing materials, the phantom could possess long‐term physical stability for quality assurance. The silicone‐based and urethane‐based materials selected for the multimodality phantom practically satisfy the requirement.

Silicone fluids, such as polydimethylsiloxane oil, have been used for simulating tissue phantoms because the mechanical properties of the phantom could be modified with the addition of silicone oil.^35^ In this study, silicone oil was added prior to curing to modify the signal intensity across various ranges. With this approach, imaging properties similar to those of the scans of soft tissue structures and inner pelvic bone of the patient from the CT and MR images were obtained. However, the polymer network can only hold a limited weight percentage of silicone oil before reaching saturation. The material used to mimic the bladder, that is, 40 wt.% silicone oil in VytaFlex, was not cured over the time reported by the manufacturer; hence, the curing time was extended. The phantom CT images indicated that air bubbles (possibly generated by the exuding silicone oil) hardened the bladder region. Nevertheless, the pixel intensities in the CT and MR of the region were not remarkably affected; the measured intensities were comparable to those of patient images.

Using the oil with a hardening agent, adipose tissue simulation was achieved; imaging characteristics similar to those of patient scans were obtained. The viscous liquid materials can completely fill all voids (i.e., no empty spaces) before hardening; hence, they are suitable for manufacturing phantoms with complex structures. However, these materials are affected by the air bubbles generated by manual stirring. Accordingly, to minimize bubble formation, the phantom was placed in a pressure chamber for material curing.

Deep learning has exhibited remarkable performance in processing large‐scale image datasets in the field of medical imaging.^36^ For the generation of sCT, various convolutional neural network methods compared with the atlas‐based method were found to be more capable of producing highly accurate sCT estimations in near real time. A key feature in deep learning techniques is not only the novel model architecture for extracting data representations but also the superior quality of the datasets processed. The consistency of the distribution between the training and test datasets is crucial to ensure reliable performance.^37^ Therefore, the geometric and imaging characteristics of the input image should be similar to those of the training datasets for the evaluation of the sCT algorithm.

Three‐dimensional printing technology has contributed to advances in phantom fabrication for clinical applications. Phantoms that are 3D printed are cost efficient and customizable in terms of using various types of materials; they are also extremely suitable for generating the complex structures of human organs. The anthropomorphic pelvic phantom in this study was designed based on the organs of a patient; hence, its use for evaluating the deep learning model trained with patient datasets would be satisfactory.

The general procedures for the performance evaluation of the deep‐learning‐based sCT generation model are as follows.^5^ First, image registration is performed to geometrically match the CT image to the MR image. We then use the MR images as input to the trained model to generate sCT images. Finally, the performance of the model is analyzed by comparing the image intensities between sCT and deformed CT. However, the dataset inherently contained image registration uncertainty. In addition, in the case of air pockets or fecal matter in the bowel that may change in position between MR and CT scans, the pixelwise difference metrics assess the region as an error. Several studies of deep‐learning‐based sCT models have tried to remove the impact of mismatch of air pockets location by assigning the region both CT and MR images with air or soft tissue or using the CycleGAN model for the unpaired datasets.^14^, ^15^, ^16^ Even if the sCT algorithm well generates the inconsistent regions between CT and MR images, the metrics have difficulty evaluating the nonrigid organs and air pockets. One of the solutions for these issues is the use of a rigid phantom resembling the patients who ensures consistency of the organ locations for deep learning model verification.

As an application of the multimodality phantom, we dosimetrically evaluated the sCT algorithm. An IMRT plan was generated based on CT_sim_; then, the same plan was copied for CT_syn_. The study results indicated that there was no remarkable clinical dosimetric difference between the two plans. However, the phantom was not designed for the dosimetric verification of the plan with MR‐compatible ion chambers or optically stimulated luminescent dosimeters. Future work can be designed with a modular phantom that incorporates inserts into the dosimeter or radiochromic film for point or planar dose verifications.

This study had several limitations. The design of the phantom structure was rescaled to fit the 3D printer size; nevertheless, no negative results in the generation of sCT images were observed. If the phantom is to be widely employed in clinical procedures, a 3D printer with a larger capacity can be used to increase the geometric similarity with the patient case. In this study, a phantom of the pelvic region was fabricated. This area was selected because its phantom was relatively simple to produce with 3D printing technology. A phantom that closely resembled the patient organs was necessary to appropriately evaluate the trained model using patient images. Recently, sCT generation algorithms have been applied to the study of the abdominal area and other regions^19^, ^38^, ^39^; therefore, more complex phantom fabrication will be necessary to evaluate the algorithm in the future. Moreover, the tissue‐surrogate materials were selected based on the measurements of the 0.35 T MR scanner with the TrueFISP sequence, which is clinically available only in the ViewRay system. If a sCT generation model was trained with images acquired from a higher Tesla MR machine, such as an Elekta Unity MR‐linac system, it would be possible to use the proposed procedure to evaluate the model in further studies.

## 5 CONCLUSION

In this study, an anthropomorphic pelvic phantom, which was physiologically and geometrically similar to the patient's organ, was fabricated using 3D printing technology; moreover, the sCT technique was evaluated quantitatively. This proposed scheme is anticipated to be useful for the quality assurance of deep‐learning‐based algorithms that use CT and MR images in radiotherapy.

## CONFLICT OF INTEREST

The authors have no relevant conflicts of interest to disclose.

## AUTHOR CONTRIBUTIONS

*Phantom design, data analysis, and writing of the paper*: Hyeongmin Jin. *Phantom design and fabrication*: Sung Young Lee. *Synthetic CT image generation*: Hyun Joon An. *Study conception and data analysis*: Chang Heon Choi. *Source of datasets and treatment planning*: Eui Kyu Chie. *Study conception and contour review*: Hong‐Gyun Wu. *Data analysis and critical review of the manuscript*: Jong Min Park. *Critical review of the manuscript*: Sukwon Park. *Study conception and supervised the project*: Jung‐in Kim. All authors discussed the results and contributed to the manuscript.

## Data Availability

The data that support the findings of this study are available from the corresponding author upon reasonable request.
